# Attitudes of nearly 7000 health professionals, genomic researchers and publics toward the return of incidental results from sequencing research

**DOI:** 10.1038/ejhg.2015.58

**Published:** 2015-04-29

**Authors:** Anna Middleton, Katherine I Morley, Eugene Bragin, Helen V Firth, Matthew E Hurles, Caroline F Wright, Michael Parker

**Affiliations:** 1 Wellcome Trust Sanger Institute, Human Genetics, Cambridge, UK; 2 Addictions Department, Institute of Psychiatry, Psychology, and Neuroscience, King's College London, London, UK; 3 Centre for Molecular, Environmental, Genetic and Analytic Epidemiology, Melbourne School of Population and Global Health, The University of Melbourne, Melbourne, Victoria, Australia; 4 Department of Clinical Genetics, Addenbrooke's Hospital, Cambridge, UK; 5 The Ethox Centre, Nuffield Department of Population Health, University of Oxford, Oxford, UK

## Abstract

Genome-wide sequencing in a research setting has the potential to reveal health-related information of personal or clinical utility for the study participant. There is increasing pressure to return research findings to participants that may not be related to the project aims, particularly when these could be used to prevent disease. Such secondary, unsolicited or 'incidental findings' (IFs) may be discovered unintentionally when interpreting sequence data, or as the result of a deliberate opportunistic screen. This cross-sectional, web-based survey investigated attitudes of 6944 individuals from 75 countries towards returning IFs from genome research. Participants included four relevant stakeholder groups: 4961 members of the public, 533 genetic health professionals, 843 non-genetic health professionals and 607 genomic researchers who were invited via traditional media, social media and professional e-mail list-serve. Treatability and perceived utility of incidental results were deemed important with 98% of stakeholders personally interested in learning about preventable life-threatening conditions. Although there was a generic interest in receiving genomic information, stakeholders did not expect researchers to opportunistically screen for IFs in a research setting. On many items, genetic health professionals had significantly more conservative views compared with other stakeholders. This finding demonstrates a disconnect between the views of those handling the findings of research and those participating in research. Exploring, evaluating and ultimately addressing this disconnect should form a priority for researchers and clinicians alike. This social sciences study offers the largest dataset, published to date, of attitudes towards issues surrounding the return of IFs from sequencing research.

## Introduction

Analysis of a genome sequence has the potential to reveal health-related information useful for disease diagnosis and prevention, as well as reams of data that is clinically irrelevant or even impossible to interpret with current knowledge.^1^ Genome-wide sequencing technologies will soon make genome analysis available to many thousands of people in clinical and research settings, for example, the Genomics England initiative plans to sequence 100 000 genomes for the National Health Service (www.genomicsengland.co.uk). Social science research that offers insight into what potential participants would want to know from their genome sequence is thus both crucial and timely.

When undertaken in response to a specific question, such as understanding the cause of a child's developmental disorder,^2^ a genome/exome sequence can be an extremely valuable tool for the discovery of pertinent or primary findings, that is, mutations in genes relevant to the disorder. However, in the absence of a specific clinical question and a relevant phenotype or family history, it is difficult to identify clinically relevant genetic variants among the thousands of variants in an individual. Even if pathogenicity of a genetic variant could be assured, and factors affecting the penetrance of the variant were clearly understood, the volume of data present considerable logistical challenges.^3^ Berg *et al*^4^ suggest that the interpretation of sequence data could be categorised into ‘bins'^4^ and only particular ‘bins' of data interrogated and returned, for example, those relating to serious, treatable conditions. The American College of Medical Genetics and Genomics recommends that sequencing a genome/exome to answer a specific clinical question provides a serendipitous opportunity to actively look for a pre-defined set of clinically relevant, non-pertinent, secondary or incidental findings (IFs).^5^

Gliwa and Berkman^6^ suggest that researchers will soon have an obligation to actively search for IFs.^6^ It is perceived as unethical not to seek information that is potentially available and could enable the recipient to undertake disease prevention measures.^6, 7, 8^ Anecdotally, genomic researchers are apprehensive about the potential impact this could have; analysing and reporting findings unrelated to the study objectives risks compromising the research endeavour. In the future, logistical difficulties relating to this may ease, in which case active searching for particular IFs could become feasible.^6^ Irrespective of the practicalities, others argue that genomic researchers have no duty (legal or otherwise) to actively search for IFs in research.^9^

The development of policy for sequencing in both research and clinical settings should take account of stakeholder views and experiences.^3, 10^ A systematic review^11^ of the psychosocial research literature in this area reveals an ‘urgent need for empirical investigations' (Jackson *et al*^11^ p. 1) because of an existing paucity of data that can inform policy. Recently, some valuable, but small-scale studies have emerged from individual countries^12, 13, 14, 15, 16, 17, 18^ on the issue of data return in both a clinical and research setting. However, as yet there have been no large international studies that gather data across multiple stakeholders from multiple countries and continents.

Relevant stakeholders include, but are not limited to: (1) members of the public, who are potential participants in sequencing studies; (2) genomic researchers (molecular scientists and bioinformaticians) who create sequencing assays and perform computer-based analysis; (3) genetic health professionals (clinical geneticists, genetic counsellors and diagnostic lab staff) – who have expertise in genetics/genomics including data interpretation and explaining results to patients, and who may be called upon to validate sequencing findings obtained in a research context; (4) non-genetic health professionals (surgeons, general physicians, nurses and midwives) – who work in a healthcare setting and may care for patients receiving results from genomic studies. As genomics moves into mainstream medical practise, there will be an increasing need for this latter group to engage with these issues.

## Materials and methods

Detailed descriptions of and justifications for the adopted survey design process, validity, readability and reliability testing and participant recruitment strategy have been published in two independent methods papers,^19, 20^ these are summarised below.

The study has UK Research Ethics Committee approval (10/H0305/83 and 11/EE/0313 granted by the Cambridge South REC).

### Survey

A cross-sectional, web-based survey (www.genomethics.org) was designed to gather international attitudes towards information arising from genome sequencing.^19^ Ten short films, embedded within the survey, were used as a medium to illustrate the ethical issues raised by genome sequencing (see Supplementary Information for an example of one of the films). In this article, categorical data are reported on the following:

1. Attitudes toward:
  - returning ‘pertinent findings' from whole-genome studies
  - eturning ‘incidental findings' from whole-genome studies
    ○receiving genetic information in different categories
  •risk perception
2. Sociodemographic information: gender, age, parents or not, geographical location, education, ethnic group, religiosity and marital status
3. Prior personal genetic testing/genome analysis and recruitment method

### Recruitment

Participants were recruited via a combination of traditional media (ie, television, radio and online news items on the research), social media (Facebook, Twitter, LinkedIn, Google Ads and a Blog) and direct e-mail invitation of professional groups comprising health professionals and genomic researchers.^20^ The recruitment strategy was designed for scale and breadth rather than to collect a representative sample. Data were collected between January 2012 and July 2013; each sampling strategy was pursued concurrently throughout this time. As this was an anonymous survey, it is not possible to investigate any overlaps in source of recruitment. However, more information can be found on the interplay between the recruitment strategy and resultant sample in one of our methods papers.^20^

### Participants

As the survey was web based and available to any English-speaking Internet user, irrespective of geography, a convenience and snowballing sampling framework was exploited.^20^ Responses to the survey were anonymous and consent was deemed implicit if participants chose to answer the questions. Participants actively invited for participation included four different stakeholder groups:

1. Members of the public
2. Genetic health professionals
3. Non-genetic health professionals
4. Genomic researchers.

Participant data are presented in two ways. Stratified responses by stakeholder groups are provided; these unadjusted data identify the significant associations between stakeholder groups (details in the Supplementary Information). Then, adjusted data provide estimates of these associations (see statistical analysis below).

### Statistical analysis

Participant responses to survey questions were summarised as percentages, calculated by stakeholder group (general public, genetic health professionals, non-genetic health professionals and genomic researchers). Unadjusted associations between items and stakeholder group were estimated using non-parametric *χ*^2^ tests. Binary logistic regression was used to provide estimates of these associations adjusted for the following covariates: age, geographical location, gender, education, ethnicity, marital status, parent or not, religiosity, previous personal involvement in genetic testing or genomic analysis, and recruitment method. All covariates were collected as categorical variables.

Latent class analysis (LCA) was used to identify subgroups with different overall attitudes to returning genomic results. The method defines subgroups (or classes) based on responses to a subset of the survey questions, which were selected for lack of redundancy and potential capacity to discriminate between subgroups. The LCA provides estimates of the proportion of the sample in each class and, for each class, the probability that class members will respond negatively to each question (item-response probabilities).^21^

Models postulating different numbers of classes were fitted and the best-fitting model identified by information criteria (Akaike information criterion and Bayesian information criterion), and consideration of the size, distinctness and ease of interpretation of the identified classes.^22, 23^ Further models were fitted to examine whether latent group membership was predicted by stakeholder group, adjusted for age, gender, education, geographical location, marital status, ethnic group and recruitment method. Statistical analyses were conducted using the software packages IBM SPSS Statistics (version 21; www.ibm.com) and SAS (version 9.3 for Windows; www.sas.com) with the PROC LCA plugin.^23^

## Results

There were 6944 participants in the sample: 4961 members of the public, 533 genetic health professionals, 843 non-genetic health professionals (eg, surgeons, general physicians, nurse and midwives) and 607 genomic researchers, recruited from 75 different countries (see Table 1). Profiles of the participants who opened the survey and then declined to complete it or provided inconsistent answers are published separately.^19^

Although participants were from 75 different countries, geography was not found to consistently nor significantly affect attitudes (data not shown). Membership of the different stakeholder groups was a more powerful indicator of attitudes and for this reason the data are presented by stakeholder group membership. Thus, irrespective of whether, for example, a genetic health professional was from the United Kingdom, United States, France, The Netherlands, Australia or the Middle East, their attitudes were more similar to each other, and unaffected by geography; this was the same for all other stakeholder groups too.

### Participant characteristics

Supplementary Table S1 in the Supplementary Information provides full details of the participant characteristics. Here, the four stakeholder groups were stratified by recruitment method, demographic data, as well as previous genetic testing/genome analysis.

### Full set of unadjusted and adjusted data

Supplementary Table S2 in the Supplementary Information provides the full, unadjusted data set of attitudes from all stakeholder groups with respect to receiving genomic information. Table 2 provides the full, adjusted data set of attitudes from all stakeholder groups with respect to receiving genomic information.

A summary of the particularly relevant findings is reported below.

#### Attitudes towards making pertinent and incidental findings (IFs) available to research participants

**Figure 1:**
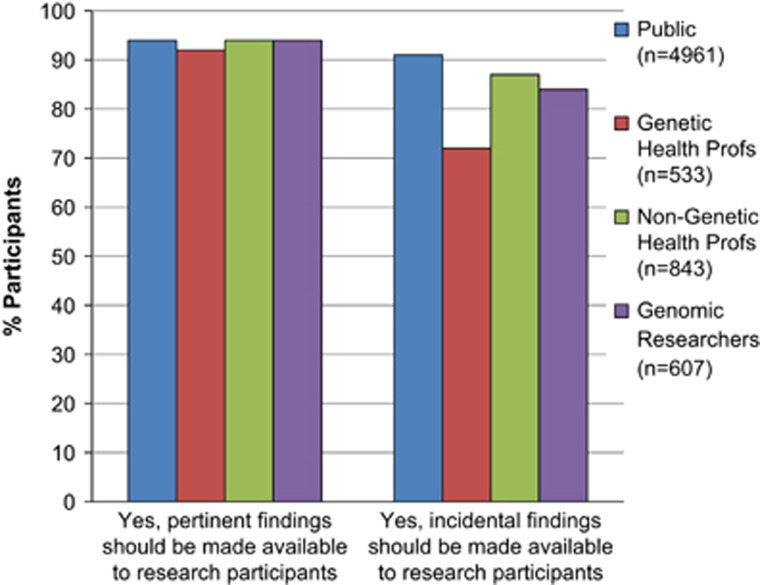
Attitudes from each stakeholder group towards making pertinent or incidental findings from genome studies available to research participants.

**Figure 2:**
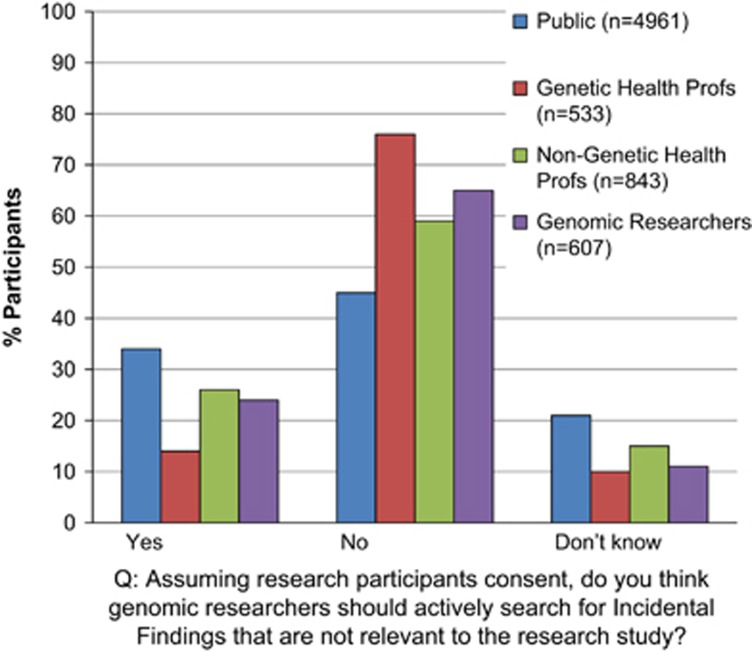
Attitudes from each stakeholder group towards opportunistic genomic screening in sequencing research.

The vast majority of all stakeholders thought that both pertinent findings and IFs from genome studies should be made available to research participants if they wanted them (Figure 1). However, when asked if genomic researchers should deliberately search for IFs that were not relevant to their research, only a minority thought this was reasonable (Figure 2) and the most likely to hold this view were the genetic health professionals (*X*^2^=229, df=6, *P*<0.0001) (Supplementary Table S2 in the Supplementary Information).

Exploring these results by stakeholder group, and adjusting for potential confounding factors (Table 2), the results show that compared with the public, genetic health professionals were five times more likely to think that IFs should not be returned (OR=5.86, CI=4.14–8.29, *P*<0.0001) and three times more likely to think that genomic researchers should not actively search for IFs irrelevant to their research (OR=3.09, CI=2.23–4.28, *P*<0.0001). Genomic researchers were also moderately more likely to feel that they should not be required to actively search for IFs irrelevant to their research (OR=1.55, CI=1.22–1.95, *P*<0.0001).

#### Attitudes towards receiving genomic information in various categories

**Figure 3:**
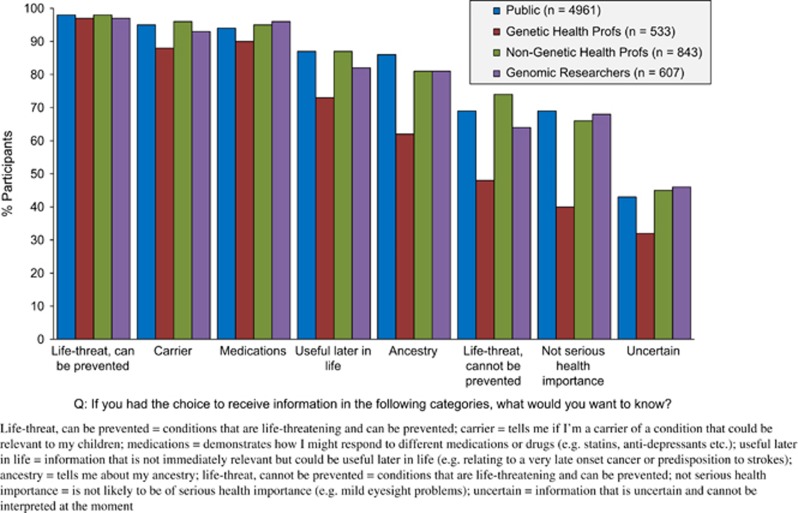
Attitudes from each stakeholder group towards receiving incidental findings in different categories.

Figure 3 shows the attitudes of each stakeholder group towards receiving genomic information in various categories. The general trend is that as the ‘severity' of the condition/information appears to decrease, so too does the positive attitude towards receiving this. In addition to this trend, participants were much more likely to be interested in information relating to life-threatening conditions if the condition was preventable, that is, treatability is important. In the adjusted data presented in Table 2 one category that shows a clear difference between stakeholder groups relates to ancestry data. Here genetic health professionals (OR=4.29, CI=3.26–5.64, *P*<0.0001) and genomic researchers (OR=1.77, CI=1.36–2.31, *P*<0.0001) are more likely, compared with the public, to believe that ancestry data should not be returned.

Significant differences exist in attitudes between stakeholder groups, especially between members of the public and genetic health professionals (Figure 3). This shows that, while they are still positive about returning information, in the main, genetic health professionals are significantly less likely to think that information in the different categories should be returned, compared with members of the public (see Supplementary Table S2 in the Supplementary Information for appropriate *P-*values in unadjusted data). These associations are still significant even when adjusting for all potential confounding effects (see Table 2 for odds ratios).

#### Attitudes towards receiving information with different levels of risk

Participants were asked to assume that it was possible to return IFs relating to a condition that is serious and preventable; and were then asked if the level of risk of actually getting the condition affected and whether they thought the result should be returned. They could choose from four levels of risk (1 in 100 or 1% 10 in 100 or 10% 50 in 100 or 50% 90 in 100 or 90%).

**Figure 4:**
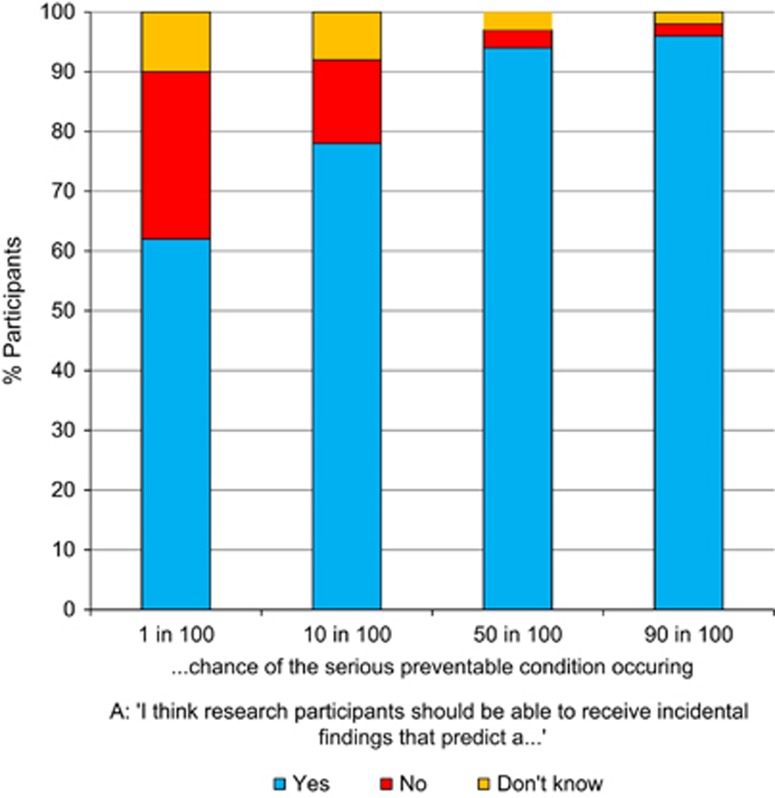
Attitudes of the whole sample towards receiving information about a serious preventable condition, which has different levels of risk.

The majority of participants thought it was acceptable to receive information in all categories, even if the risk of the condition occurring was low. As the risk increased, there was less reticence about this (ie, the no and don't know answers decreased; see Figure 4). There were no strong associations between stakeholder group and answers to this question and so this is presented in Figure 4 as attitudes of the whole sample (a breakdown by stakeholder group can be found in Supplementary Table S2 in the Supplementary Information).

#### Overall attitudes to returning genomic data

Five questions were identified for inclusion in the LCA of attitudes to returning genomic data:

1. Should IFs from genome studies be made available to research participants? [IncFind].
2. If you had the choice to receive information about conditions that are life threatening and cannot be prevented, would you want to know? [LifeCannotPr].
3. If you were a research participant in a whole-genome study, would you want to be able to receive all of your raw genomic data? [RawData] Item included in the LCA only, no other results relating to this are presented in this publication.
4. ‘If I was a research participant, I'd like to receive information that predicts a 1 in 100 risk (ie, 1% chance) that a serious preventable condition will occur' [Risk1in100].
5. ‘If I was a research participant, I'd like to receive information that is uncertain and cannot be interpreted at the moment' [Uncertain].

Models with two to five classes were fitted to the data, with the three-class model providing the best solution according to all criteria (Supplementary Table S3). Item response probabilities for each question by latent class are shown in Supplementary Table S4 in the Supplementary Information. The class membership probabilities were: 0.53 for latent class 1, 0.11 for latent class 2 and 0.36 for latent class 3.

**Figure 5:**
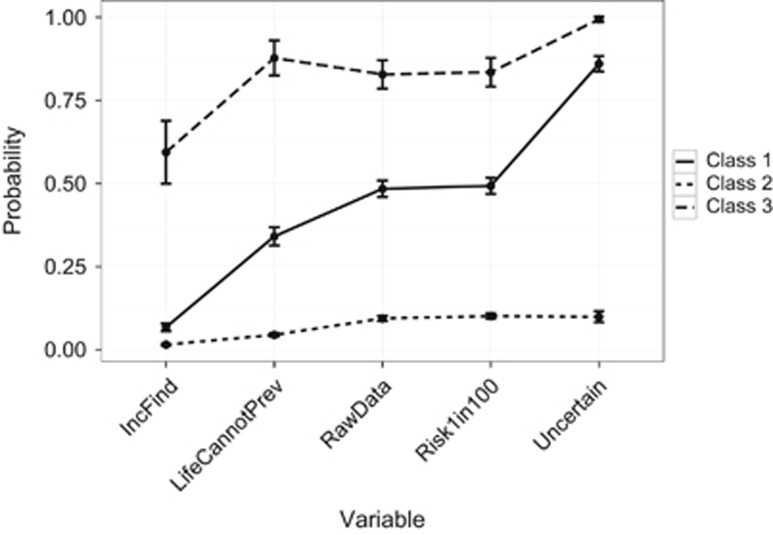
Item response probabilities (negative response) with standard errors for each latent class, for each of the questions included in the LCA.

The three underlying classes can be characterised by a pattern of responses to the five questions. The item-response probabilities depicted in Figure 5 provide a guide for interpreting each class. The largest subgroup was latent class 1, making up 53% of the sample and characterised by very low probabilities of responding negatively to the selected questions, can be labelled as having liberal attitudes to return of results. The smallest subgroup was latent class 2 (11% of the sample). Class members have high probabilities of responding negatively to the questions, and can be labelled as having negative or conservative attitudes to return of results. The remaining proportion of the sample (36%) belong to latent class 3, characterised by a very low probability of responding negatively to receiving IFs, but a very high probability of responding negatively to receiving uncertain findings. Inclusion of missing responses for the manifest variables did not alter the model selected, or substantially alter the model estimates (data not shown).

We investigated the relationship between stakeholder group and class membership, adjusting for other covariates (Table 3). We found that, compared with the general public, genetic health professionals had substantially greater odds of belonging to the negative/conservative class (latent class 2: OR=7.21, 95% CI=5.19–10.03). Genomic researchers also had slightly greater odds of belonging to this class (OR=2.29, 95% CI=1.61–3.26), but odds of membership did not differ between the public and health professionals not working in the field of genetics (OR=1.01, 95% CI=0.67–1.50). Thus, individuals who work with genetic data in clinical or research settings, especially genetic health professionals, were more likely than the general public to have a consistently negative response across a range of genomic results returning scenarios. In contrast, there was no difference between health professionals who do not regularly work with genetic data and the general public in this regard.

## Discussion

We gathered attitudes from four distinct groups of stakeholders towards various issues surrounding the receipt and return of genomic data from genome-wide sequencing research. Substantial differences in attitudes toward the return of genomic data were observed, the most extreme differences being found between members of the public and genetic health professionals. This was found to be the case when individual questions were considered separately, or when examining overall attitudes toward returning genomic data. Across most of the questions, non-genetic health professionals have attitudes that broadly align themselves with the public. This is also the case for genomic researchers too, apart from with regard to two specific issues – both genetic health professionals and genomic researchers agree that ancestry is not a category of data for which it is appropriate to search for and share. They also believe that genomic researchers should not actively search for IFs irrelevant to their research.

Four main discussion points can be drawn from this research; these are explored below.

### Attitudes are generally positive towards the concept of returning results to research participants from sequencing research

In a hypothetical scenario, potential recipients of sequencing research (public), professionals involved in running sequencing studies (genomic researchers) and associated health professionals believe that research participants should be able to receive their individual results. These include access to both primary/pertinent and secondary/unsolicited/IFs, if the participant so chooses. Genomic data thus have a perceived value to people, this appears to hold true even if that information has an uncertain or unknown interpretation. It could be that participants feel that, even if the information is uncertain in the present, it could become useful in the future. They may also feel that it just ‘belongs' to them. ‘Acquiring information is to be desired not merely for its instrumental value (ie, ‘doing something' about a potential threat), but also for its emotional value (eg, feeling assured that the threat is not imminent)'.^24^

### Treatability and perceived utility of the information is important

Participants were more interested in learning about conditions that were preventable and less interested in receiving information that is uncertain and cannot be interpreted at the moment. Returning non-health-related data, such as that associated with ancestry, while interesting for some, is not supported by genetic health professionals or genomic researchers. This suggests that the perceived utility of the data are relevant to views about feedback. Such utility is important, irrespective of how small the chance was of the condition occurring – in the question that asked whether participants would want to know about a serious, preventable condition, the majority of all participants were still interested in knowing, even if the chance of it occurring were only 1%. This fits with the findings from other empirical research,^25^ as well as ethical review^26^ that the treatability and the potential for prevention are critical. The corollary of this is that, for those people who want to make a distinction between the types of information they receive, there needs to be caution in returning information with which the recipient can do nothing in terms of health prevention.

### Some genetic health professionals have conservative attitudes toward the return of genomic data

We identified three subgroups in our sample with different overall attitudes toward the return of genomic data, one of which was characterised as having conservative views about the return of results. Genetic health professionals had the most conservative views compared with the general public. This finding demonstrates a disconnect between the views of those handling the findings of research and those participating in research. Exploring, evaluating and ultimately addressing this disconnect should form a priority for researchers and clinicians alike. Research participants appear to want access to more information than genetic health professionals consider is appropriate. Any interpretation of these findings is inevitably going to be speculative to some degree. However, as genetic health professionals are very familiar with returning results related to highly penetrant, serious, life-threatening conditions, it is unlikely that they are only concerned about returning bad news or feel that research participants should be protected from this in some way. But precisely because of this experience, they are perhaps more likely to be more cognisant than most of the importance of information being accurate and truly predictive of disease, as they routinely explain such results to patients. Thus, one of their concerns about genomic data may relate to clinical validity and utility. As we collectively understand more about normal population variation and interpretation processes improve it will become easier to determine if a suspected pathogenic variant is indeed pathogenic. And at that point it will be interesting to see whether attitudes of genetic health professionals change. Genetic health professionals as a group are notable for their patient-centred approach to care^27^ where autonomy and shared decision making are fundamental to practice.^28^ Yet qualitative research has shown that genetic health professionals feel a duty to protect their patients from potential harm caused by IFs: ‘This paternalism expressed by the geneticists' group could be seen as an attempt to keep the box closed (or to somewhat regulate its opening) and prevent the regret that the mythical Pandora felt after opening the box; essentially the duty to do no harm' (Townsend *et al*^29^ p. 2524).

### Genomic researchers should not actively search for IFs irrelevant to their research

We have shown in our data that all stakeholders support the return of IFs to research participants. However, when we asked them to consider situations where providing this information in a research setting could compromise the ability to address the primary research question, stakeholder views then shifted considerably. Participants appreciated the potential burden that managing IFs could place on genomic researchers. ‘Research, even in a clinical setting, differs from clinical care in both its goals and procedures' (Jarvik *et al*^30^ p. 819) and ‘Resources for research should be primarily directed at scientific discovery' (Jarvik *et al*^30^ p. 820). A contract exists between researcher and participant, whereby the terms of the research are presented (eg, return of results within certain boundaries) and the research participant can choose to take part under those conditions or decline participation.

Genetic health professionals may assume that if the research participant does not agree with the protocol for result feedback that they can ‘vote with their feet' and decline participation. However, for many patients, for example, with a rare genetic disorder in themselves or their child, there may be an inherent clinical motivation to participating in research; in the absence of clinical sequencing being available widely across all areas of mainstream medicine, a research study may provide the only current opportunity to receive a diagnosis. For patients considering whether to participate in a sequencing study, it may not be as simple as ‘take it or leave it' and in many instances patients may not be able to access a genetic diagnosis for themselves or their children in any other way.

Further speculation on the views of genetic health professionals leads us to consider the perceived burden of whole-genome sequencing on their time and role. It is possible that they anticipate an enormous increase in workload and feel ill-prepared to tackle the challenge of variant interpretation related to clinical diagnosis, let alone variant interpretation of IFs. As genomics is mainstreamed and more healthcare professionals outside specialist clinical genetics services order sequencing tests, the referral rate back to genetic health professionals may increase if non-genetics health professionals need support in order to understand and interpret genomic laboratory reports or with counselling expertise. Returning IFs implies that it is possible to interpret the results clinically, which may not be the case where the evidence-based is sparse. Returning IFs from research overlays more complexity and an additional burden for associated clinicians to manage.

Recent policy recommendations from genetic health professionals, for example, the European Society of Human Genetics, and the Association of Genetic Nurses and Counsellors in the United Kingdom and Ireland highlight the reality, and uncertainty of working with genomic data.^10, 31^ However, participants with a negative overall attitude towards the questions selected for the LCA represented the smallest subgroup in our sample (making up approximately 11%), and the majority of genetic health professionals responded positively to specific questions regarding the return of pertinent and IFs.

### Development of policy

Although the empirical data from this research show that all four stakeholder groups are in favour of returning genomic results of perceived utility to research participants, we also show that when pushed, no stakeholder group actually expects these to be provided in a research setting if doing so would compromise the ability of researchers to answer their research question. Hence, policy does not need to obligate researchers to return IFs from research sequencing studies.^32^ Policy development in this area should take into account not only the empirical data from stakeholders, as well as the ethical debate, but also other compelling practical considerations – most notably, the confidence (or lack thereof) with which such variant data can be accurately interpreted and the implications for successful and financially viable research.

As our data have shown, there is support across all of our stakeholder groups for the return of IFs in a research setting, even if these are not actively searched for; and although our participants appreciate that there may be a practical burden on researchers and health professionals to manage this (and indeed they say they will forgo their results if the searching for them is too burdensome), it is nevertheless the case that they subscribe in principle to the return of results. This suggests that policy makers, funders, genomic researchers and health professionals should consider how to address the hurdles that obstruct this process.

The Presidential Commission for the Study of Bioethical Issues argues that ‘Prioritizing a duty to look for secondary findings over the creation of generalisable knowledge has the potential to undermine the research enterprise' (Presidential Commission^9^ p. 91). However, this group also acknowledge that researchers may choose to seek funding specifically so that they can perform opportunistic screening. If it was straightforward to adapt pipelines, for example, to screen for a pre-determined list of health-related variants, then opportunistic genomic screening in a research setting may become feasible but it would require careful evaluation to determine the relative benefits, harms and costs of such an intervention.

Unless (opt out) opportunistic screening becomes part of routine clinical sequencing, as per the recommendations from the American College of Medical Genetics and Genomics (ACMG),^5^ it would be inappropriate to introduce clinical-grade opportunistic screening in a research setting before it has been fully evaluated to determine the context and circumstances in which such intervention may be beneficial. Despite gathering momentum in the United States, the enthusiasm for opportunistic genomic screening in Europe has thus far been lacking. European policy makers support the use of targeted sequencing to answer a specific clinical question and suggest that scientists try to avoid discovering unsolicited [incidental] findings.^31, 33^ A situation may therefore arise with clinical sequencing where in some parts of the world (eg, United States), opportunistic genomic screening becomes part of practice where others (eg, United Kingdom and other European countries, Australasia) may favour a more cautious approach.

### Limitations

The recruitment strategies were deliberately designed to enable the collection of a large, international sample, but the convenience and snowballing sampling framework meant that it would never be possible for the final sample to be considered representative of any particular group. In our methods paper on the recruitment strategy, we describe the biases that exist in the study sample.^20^ We also demonstrate how the participant profile is very similar to those from other social sciences research about genetics and thus the biases present are typical of many other studies too. Endeavours have been made to minimise the biases as much as possible by adjusting for potential confounding factors in analyses, but the results described here cannot be generalised and should be viewed as a starting point for further research in this area. Using an online survey means that there are no details on non-response rate.

Although this research provides valuable evidence about the prevalence of views about and attitudes towards the hypothetical return of IFs, what stakeholders do in a real situation may be different. Attitudes are thought to be the one of the best predictors of behaviour,^34^ but until IFs are returned in reality, and the experience of this is measured, it is impossible to know how closely these are aligned. Although the development of policy on IFs should be informed by robust evidence, the question of what to do in individual cases will always need to take into account the beliefs and preferences of individual research participants.^9, 10, 33^ Furthermore, although it appears clear that the majority of stakeholders believe the feedback of IFs to be a positive thing, this does not necessarily mean that this is the most appropriate policy to adopt now. It may be, but it may also be that other considerations or arguments that also impact on this question, for example, the appropriate use of limited health care or research resources and difficulties in data interpretation, result in a different conclusion.

A further limitation of the study is that participants were not asked for their views on returning IFs from other, non-genomic, medical research.

### Summary

The medical, genetics, ethics and social sciences literature currently includes debate about the pros and cons of returning genomic data in a research and clinical setting^5, 35, 36^ and whether there should be a requirement to search for and feedback IFs from whole-genome studies to individual participants.^7, 19, 37^ ‘Even pure scientists can and should advance research subjects well-being and respect their autonomy by making appropriate disclosures of potentially significant Ifs' (Miller *et al*^38^ p. 278). In this article, we report on a large-scale empirical research study that explored attitudes across all stakeholder groups towards the return of IFs from sequencing research. We found that the biggest factor affecting attitudes was whether participants were members of the public, genetic health professionals, non-genetic health professionals or genomic scientists. Where the participant was from, geographically speaking, in the world, was not significant. We have found that although there is a generic interest in receiving genomic information, stakeholders do not expect this to be provided in a research setting when doing so is not directly related to the aims of the research. It is our hope that this evidence, and other empirical evidence relating to this issue, will inform the development of policy on the future use of genomics in research and clinical practice.

## Figures and Tables

**Table 1:** Members within each stakeholder group

*Public (n=4961)*
2% (*n*=158) Have taken part in biobank research; 3% (*n*=694) have had genetic testing or sequencing before; 83% (*n*=4109) have had no involvement with genetics before
*Genetic health professionals (n=533)*
26% (*n*=136) Clinical geneticist; 26% (*n*=140) genetic counsellor; 33% (*n*=176) diagnostic lab scientist; 15% (*n*=81) genomic researcher and health professional
*Non-genetic health professional (n=843)*
22% (*n*=185) Doctor (eg, GP, radiologist, surgeon, oncologist, paediatrician, med student); 57% (*n*=483) professionals allied to medicine (eg, nurse, dietician, occupational therapist, ambulance driver, clinical trials etc); 21% (*n*=175) medical administrators, ethics committee member, health researcher
*Genomic researcher (n=607)*
44% (*n*=267) Computer-based scientist (ie, bioinformatician); 41% (*n*=249) lab scientist (inc Head/PI); 15% (*n*=91) other (eg, policy, education, social scientist, ethicist, public health)

**Table 2:** Attitudes towards receiving genomic data: adjusted^a^ results

*Question*	*Public*	*Genetic health profs*	*Non-genetic health profs*	*Genomic researchers*	*Total* *N*
***Should pertinent findings from genome studies be made available to research participants?***	Ref. 1	2.78^b^ (1.43–5.42)	1.99 (0.98–4.08)	1.81 (0.98–3.37)	4691
0=Research participants should be able to choose to receive pertinent findings, if they want them; 1=I don't think pertinent findings from research projects should be available; Don't know responses not included in analysis		*P*=0.003	*P*=0.06	*P*=0.06	
***Should incidental findings from genome studies be made available to research participants?***	Ref. 1	5.86 (4.14–8.29)	2.72 (1.83–4.04)	1.52 (1.01–2.29)	4618
0=Research participants should be able to choose to receive incidental findings, if they want them; 1=I don't think pertinent findings from research projects should be available; Don't know responses not included in analysis		*P*<0.0001	*P*<0.0001	*P*=0.04	
***Let's imagine you are a research participant. If you had the choice to receive information in the following categories, what would you want to know? ‘I'd like to know about...' …*conditions that are life threatening and cannot be prevented** 0=Yes, 1=no, don't know responses not included in the analysis	Ref. 1	3.84 (2.95–5.01) *P*<0.0001	2.02 (1.54–2.65) *P*<0.0001	0.92 (0.71–1.20) *P*=0.55	4151
**…conditions that are life threatening and can be prevented**	Ref. 1	1.76 (0.71–4.35)	1.12 (0.42–2.99)	1.35 (0.54–3.38)	4176
		*P*=0.22	*P*=0.82	*P*=0.52	
**…conditions that are serious (but not life threatening) and cannot be prevented**	Ref. 1	5.65 (4.30–7.42)	2.10 (1.57–2.81)	1.28 (0.98–1.67)	4309
		*P*<0.0001	*P*<0.0001	*P*=0.07	
**…conditions that are serious (but not life threatening) and can be prevented**	Ref. 1	2.33 (1.05–5.14)	1.31 (0.52–3.27)	2.30 (1.11–4.76)	4703
		*P*=0.04	*P*=0.57	*P*=0.03	
***‘If I was a research participant, I'd like to receive information that...'* …demonstrates how I might respond to different medications or drugs (eg, statins, anti-depressants etc)**	Ref. 1	2.10 (1.26–3.49) *P*=0.005	0.77 (0.39–1.53) *P*=0.46	1.50 (0.93–2.43) *P*=0.10	4627
0=Yes, 1=no, don't know responses not included in the analysis					
**.. tells me if I'm a carrier of a condition that could be relevant to my children**	Ref. 1	2.54 (1.55–4.18)	1.08 (0.59–2.00)	0.76 (0.41–1.42)	4645
		*P*<0.0001	*P*=0.81	*P*=0.39	
**.. is not immediately relevant but could be useful later in life (eg, relating to a very late onset cancer or predisposition to strokes)**	Ref. 1	3.67 (2.63–5.13) *P*<0.0001	1.86 (1.28–2.70) *P*=0.001	1.36 (0.96–1.93) *P*=0.08	4468
**.. is uncertain and cannot be interpreted at the moment**	Ref. 1	1.98 (1.52–2.56)	1.12 (0.89–1.42)	0.95 (0.77–1.16)	4068
		*P*<0.0001	*P*=0.33	*P*=0.60	
**... is not likely to be of serious health importance (eg, mild eyesight problems)**	Ref. 1	3.67 (2.86–4.71)	1.06 (0.82–1.36)	1.10 (0.89–1.36)	4533
		*P*<0.0001	*P*=0.67	*P*=0.36	
**.. tells me about my ancestry**	Ref. 1	4.29 (3.26–5.64)	1.18 (0.84–1.66)	1.77 (1.36–2.31)	4572
		*P*<0.0001	*P*=0.33	*P*<0.0001	
***Let's assume it is possible to return incidental findings relating a condition that is serious and preventable. Does the level of risk of actually getting the condition affect whether you think the result should be returned? ‘If I was a research participant, I'd like to receive information that predicts...'* there is a 1 in 100 risk (ie, 1% chance) that this condition will occur** 0=Yes, 1=no, don't know responses not included in the analysis	Ref. 1	1.34 (1.04–1.72) *P*=0.02	0.81 (0.63–1.05) *P*=0.11	0.68 (0.54–0.85) *P*=0.001	4282
**... there is a 10 in 100 risk (ie, 10% chance) that this condition will occur**	Ref. 1	1.28 (0.93–1.75)	1.21 (0.89–1.64)	0.83 (0.62–1.10)	4389
		*P*=0.14	*P*=0.23	*P*=0.20	
**... there is a 50 in 100 risk (ie, 50% chance) that this condition will occur**	Ref. 1	0.99 (0.51–1.94)	1.48 (0.84–2.60)	0.91 (0.51–1.61)	4635
		*P*=0.98	*P*=0.18	*P*=0.74	
**... there is a 90 in 100 risk (ie, 90% chance) that this condition will occur**	Ref. 1	0.60 (0.18–2.02)	1.95 (0.93–4.09)	1.31 (0.63–2.73)	4673
		*P*=0.41	*P*=0.08	*P*=0.47	
***Assuming research participants consent, do you think genomic researchers should actively search for incidental findings that are not relevant to the research study?***	Ref. 1	3.09 (2.23–4.28) *P*<0.0001	1.50 (1.15–1.95) *P*=0.003	1.55 (1.22–1.95) *P*<0.0001	3944
0=Yes, 1=no, don't know responses not included in the analysis					

a Adjusting for: gender, age, geography, education, ethnicity, religiosity, marital status, parent or not, recruitment method, previous genetic testing/genomic analysis.

b Odds ratios with 95% confidence intervals in brackets.

**Table 3:** Odds ratios and 95% confidence intervals for class membership by stakeholder group, adjusted for gender, age, education, country of residence, marital status, ethnicity and recruitment method

*Stakeholder group*	*Latent class 2 (conservative)*		*Latent class 3 (intermediate)*	
	*OR*	*95% CI*	*OR*	*95% CI*
Public	Ref.		Ref.	
Genomic researchers	2.29	(1.61–3.26)	0.90	(0.73–1.11)
Genetic health professionals	7.21	(5.19–10.03)	1.56	(1.22–2.00)
Non-genetic health professionals	1.01	(0.67–1.50)	1.08	(0.90–1.29)

## Supplementary Material

Supplementary Tables

An example of the ten films integrated into the survey
