# Supplementary material for: Attitudes of nearly 7000 health professionals, genomic researchers and publics toward the return of incidental results from sequencing research
Source: Eur J Hum Genet. 2015 Apr 29;24(1):21–9. doi: 10.1038/ejhg.2015.58 (PMC4795240; doi:10.1038/ejhg.2015.58)
Supplement: Supplementary Tables [file ejhg201558x1.doc]

Supplementary Information

| Table S1: Participant characteristics | | | | | |
| --- | --- | --- | --- | --- | --- |
| Question | Public  (n=4961) | Genetic Health Profs  (n = 533) | Non-Genetic Health Profs  (n = 843) | Genomic Resear-chers  (n = 607) | Total  (n=6944) |
| Recruitment method |  |  |  |  |  |
| Social media Observed (Expected)  % Within Column | 3048  (2992)  79% | 232  (306)  58% | 492  (455)  82% | 276  (365)  57% | 4048  76% |
| Direct invitation Observed  (Expected)  % Within Column | 402  (604)  10% | 166  (63)  41% | 75  (94)  13% | 193  (76)  40% | 836  16% |
| Traditional media Observed  (Expected)  % Within Column | 404  (328)  11% | 5  (34)  1% | 33  (51)  5% | 13  (41)  3% | 455  8% |
| Total | 3854  100% | 403  100% | 600  100% | 482  100% | 5339  100% |
| Missing values | 1107 | 130 | 243 | 125 | 1605 |
| Gender |  |  |  |  |  |
| Male Observed  (Expected)  % Within Column | 932  (1010)  24% | 117  (110)  28% | 125  (159)  20% | 234  (129)  47% | 1408  26% |
| Female Observed  (Expected)  % Within Column | 2962  (2878)  75% | 302  (312)  71% | 486  (453)  79% | 262  (369)  52% | 4012  73% |
| Prefer not to say Observed  (Expected)  % Within Column | 19  (24)  1% | 5  (3)  1% | 5  (4)  1% | 5  (3)  1% | 34  1% |
| Total | 3913  100% | 424  100% | 616  100% | 501  100% | 5454  100% |
| Missing values | 1048 | 109 | 227 | 106 | 1490 |
| Age |  |  |  |  |  |
| Under 16 Observed  (Expected)  % Within Column | 53  (38)  1% | 0  (4)  0% | 0  (6)  0% | 0  (5)  0% | 53  1% |
| Age 17-20 Observed  (Expected)  % Within Column | 169  (129)  4% | 0  (14)  0% | 8  (20)  1% | 3  (17)  1% | 180  3% |
| Age 21-30 Observed  (Expected)  % Within Column | 737  (843)  19% | 101  (91)  24% | 130  (133)  21% | 207  (108)  41% | 1175  21% |
| Age 31-40 Observed  (Expected)  % Within Column | 1172  (1185)  30% | 142  (128)  34% | 172  (187)  28% | 165  (151)  33% | 1651  30% |
| Age 41-50 Observed  (Expected)  % Within Column | 758  (800)  19% | 102  (87)  24% | 165  (126)  27% | 90  (102)  18% | 1115  21% |
| Age 51-60 Observed  (Expected)  % Within Column | 506  (492)  13% | 63  (53)  15% | 91  (77)  15% | 25  (63)  5% | 685  13% |
| Age 61-70 Observed  (Expected)  % Within Column | 406  (340)  10% | 14  (37)  3% | 45  (54)  7% | 9  (44)  2% | 474  9% |
| Age 71-80 Observed  (Expected)  % Within Column | 104  (80)  3% | 1  (9)  0.2% | 5  (13)  1% | 1  (10)  0.2% | 111  2% |
| Over 81+ Observed  (Expected)  % Within Column | 8  (6)  1% | 0  (1)  0% | 0  (1)  0%) | 0  (1)  0% | 8  0.1% |
| Total | 3913  100% | 423  100% | 616  100% | 500  100% | 5452  100% |
| Missing values | 1048 | 110 | 227 | 107 | 1492 |
| Where do you live? |  |  |  |  |  |
| UK, Ireland Observed  (Expected)  % Within Column | 3911  (3623)  79% | 305  (390)  57% | 518  (616)  62% | 331  (436)  56% | 5065  73% |
| USA, Canada, Mexico Observed  (Expected)  % Within Column | 585  (767)  12% | 116  (83)  22% | 234  (130)  28% | 137  (92)  23% | 1072  16% |
| Australia, New Zealand Observed  (Expected)  % Within Column | 60  (76)  1% | 15  (8)  3% | 21  (13)  3% | 10  (9)  2% | 106  1% |
| Mainland Europe Observed  (Expected)  % Within Column | 303  (370)  6% | 67  (40)  13% | 50  (63)  6% | 97  (45)  16% | 517  8% |
| Middle East Observed  (Expected)  % Within Column | 9  (17)  0.2% | 9  (2)  2% | 1  (3)  0.1% | 5  (2)  1% | 24  0.3% |
| South America Observed  (Expected)  % Within Column | 22  (24)  0.4% | 5  (3)  1% | 5  (4)  1% | 2  (3)  0.3% | 34  1% |
| Asia Observed  (Expected)  % Within Column | 23  (37)  0.5% | 8  (4)  1% | 8  (6)  1% | 13  (5)  2% | 52  1% |
| Africa Observed  (Expected)  % Within Column | 37  (37)  1% | 8  (4)  1% | 5  (6)  1% | 1  (4)  0.2% | 51  0.7% |
| Total | 4950  100% | 533  100% | 842  100% | 596  100% | 6921  100% |
| Missing values | 11 | 0 | 1 | 11 | 23 |
| Select the option that best describes your formal education |  |  |  |  |  |
| Completed primary school/ Observed  preparatory school/elementary school (Expected)  % Within Column | 76  (60)  2% | 0  (7)  0% | 6  (9)  1% | 1  (8)  0% | 83  2% |
| Currently studying at secondary school/ Observed  high school (Expected)  % Within Column | 72  (53)  2% | 1  (6)  0% | 1  (8)  0.2% | 0  (7)  0% | 74  1% |
| Completed secondary school/high school Observed  (Expected)  % Within Column | 681  (516)  17% | 3  (56)  1% | 32  (81)  5% | 3  (66)  1% | 719  13% |
| Currently studying at university/ Observed  college/other institution (Expected)  % Within Column | 402  (349)  10% | 4  (38)  1% | 50  (55)  8% | 30  (45)  6% | 486  9% |
| Completed degree(s) at university/ Observed  college/other tertiary education institution (Expected)  % Within Column | 2499  (2725)  64% | 392  (295)  92% | 486  (429)  79% | 421  (349)  84% | 3798  70% |
| Other Observed  (Expected)  % Within Column | 183  (211)  5% | 24  (23)  6% | 41  (33)  7% | 46  (27)  9% | 294  5% |
| Total | 3913  100% | 424  100% | 616  100% | 501  100% | 5454  100% |
| Missing values | 1048 | 109 | 227 | 106 | 1490 |
| Which ethnic group do you belong to? |  |  |  |  |  |
| White Observed  (Expected)  % Within Column | 3628  (3569)  93% | 366  (388)  85% | 557  (562)  90% | 423  (455)  85% | 4974  91% |
| Afro-European, African-American, Black Observed  (Expected)  % Within Column | 38  (37)  1% | 7  (4)  2% | 5  (6)  1% | 2  (5)  0.4% | 52  1% |
| Hispanic Observed  (Expected)  % Within Column | 31  (43)  1% | 3  (5)  1% | 12  (7)  2% | 14  (6)  3% | 60  1% |
| South Asian Indian, Pakistani Observed  (Expected)  % Within Column | 53  (80)  1% | 19  (9)  5% | 15  (13)  2% | 25  (10)  5% | 112  2% |
| East Asian Chinese, Japanese Observed  (Expected)  % Within Column | 26  (43)  1% | 7  (5)  2% | 8  (7)  1% | 19  (6)  4% | 60  1% |
| Arabic, Central Asian Observed  (Expected)  % Within Column | 17  (22)  0.4% | 7  (2)  2% | 3  (3)  1% | 3  (3)  0.6% | 30  1% |
| Other Observed  (Expected)  % Within Column | 118  (117)  3% | 16  (13)  4% | 16  (18)  3% | 13  (15)  3% | 163  3% |
| Total | 3911  100% | 425  100% | 616  100% | 499  100% | 5451  100% |
| Missing values | 1050 | 108 | 227 | 108 | 1493 |
| Marital status |  |  |  |  |  |
| Married/civil partnership/living together Observed  (Expected)  % Within Column | 2713  (2723)  69% | 322  (296)  75% | 441  (429)  72% | 319  (347)  64% | 3795  70% |
| Divorced Observed  (Expected)  % Within Column | 218  (194)  6% | 16  (21)  4% | 28  (31)  4% | 9  (25)  1% | 271  5% |
| Separated Observed  (Expected)  % Within Column | 63  (61)  2% | 6  (7)  1% | 13  (10)  2% | 3  (8)  1% | 85  1% |
| Widowed Observed  (Expected)  % Within Column | 81  (63)  2% | 2  (7)  1% | 4  (10)  1% | 1  (8)  0.2% | 88  2% |
| Single Observed  (Expected)  % Within Column | 836  (870)  21% | 79  (95)  19% | 130  (137)  21% | 167  (111)  34% | 1212  22% |
| Total | 3911  100% | 425  100% | 616  100% | 499  100% | 5451  100% |
| Missing values | 1050 | 108 | 227 | 108 | 1493 |
| Do you have children? |  |  |  |  |  |
| Yes Observed  (Expected)  % Within Column | 2610  (2430)  67% | 229  (263)  54% | 374  (383)  61% | 174  (311)  35% | 3387  62% |
| No Observed  (Expected)  % Within Column | 1303  (1483)  33% | 195  (161)  46% | 242  (234)  39% | 327  (190)  65% | 2067  38% |
| Total | 3913  100% | 424  100% | 616  100% | 501  100% | 5454  100% |
| Missing values | 1048 | 109 | 227 | 106 | 1490 |
| Independently of whether you attend religious services or not, would you say you are… |  |  |  |  |  |
| …a religious person Observed  (Expected)  % Within Column | 1137  (1152)  29% | 133  (125)  31% | 234  (181)  38% | 101  (147)  20% | 1605  29% |
| …not a religious person Observed  (Expected)  % Within Column | 2774  (2759)  71% | 292  (300)  69% | 382  (435)  62% | 398  (352)  80% | 3846  71% |
| Total | 3911  100% | 425  100% | 616  100% | 499  100% | 5451  100% |
| Missing values | 1050 | 108 | 227 | 108 | 1493 |
| Have you personally had genetic testing or genomic analysis done before? |  |  |  |  |  |
| Yes Observed  (Expected)  % Within Column | 577  (663)  15% | 117  (72)  27% | 88  (104)  14% | 142  (85)  28% | 924  17% |
| No Observed  (Expected)  % Within Column | 3212  (3146)  82% | 304  (341)  72% | 515  (495)  84% | 354  (403)  71% | 4385  80% |
| Don’t know Observed  (Expected)  % Within Column | 124  (104)  3% | 3  (11)  1% | 13  (16)  2% | 5  (13)  1% | 145  3% |
| Total | 3913  100% | 424  100% | 616  100% | 501  100% | 5454  100% |
| Missing values | 1048 | 109 | 227 | 106 | 1490 |

| Table S2: Attitudes towards receiving genomic data: unadjusted results | | | | | |
| --- | --- | --- | --- | --- | --- |
| Question | Public    (n= 961) | Genetic Health Profs  (n = 533) | Non-Genetic Health Profs  (n = 843) | Genomic Resear-chers  (n = 607) | Total  (n=6944) |
| Should pertinent findings from genome studies be made available to research participants? |  |  |  |  |  |
| Research participants should be able Observed  to receive pertinent findings, (Expected)  if they want them % Within Column | 4333  (4321)  94% | 470  (482)  92% | 761  (762)  94% | 550  (549)  94% | 6114  94% |
| I don’t think pertinent findings Observed  from research projects should be available (Expected)  % Within Column | 88  (107)  2% | 22  (12)  4% | 21  (19)  3% | 21  (14)  4% | 152  2% |
| I don’t know Observed  (Expected)  % Within Column | 183  (175)  4% | 21  (20)  4% | 30  (31)  4% | 14  (22)  2% | 248  4% |
| X^2^ = 20, df = 6, P = 0.003 Total | 4604  100% | 513  100% | 812  100% | 585  100% | 6514  100% |
| Missing values | 357 | 20 | 31 | 22 | 430 |
| Should incidental findings from genome studies be made available to research participants? |  |  |  |  |  |
| Research participants should be able Observed  to receive incidental findings, (Expected)  if they want them % Within Column | 4085  (3965)  91% | 365  (449)  72% | 691  (702)  87% | 487  (512)  84% | 5628  88% |
| I don’t think incidental findings Observed  from research projects should be available (Expected)  % Within Column | 198  (299)  4% | 101  (34)  20% | 55  (53)  7% | 70  (39)  12% | 424  7% |
| I don’t know Observed  (Expected)  % Within Column | 205  (224)  5% | 42  (25)  8% | 48  (40)  6% | 23  (29)  4% | 318  5% |
| X^2^ = 229, df = 6, P < 0.0001 Total | 4488  100% | 508  100% | 794  100% | 580  100% | 6370  100% |
| Missing values | 473 | 25 | 49 | 27 | 574 |
| Let's imagine you are a research participant. If you had the choice to receive information in the following categories, what would you want to know?  "I'd like to know about ..." …conditions that are life-threating and cannot be prevented |  |  |  |  |  |
| Yes Observed  (Expected)  % Within Column | 2951  (2892)  69% | 212  (295)  48% | 478  (436)  74% | 326  (344)  64% | 3967  67% |
| No Observed  (Expected)  % Within Column | 741  (846)  17% | 173  (86)  39% | 105  (128)  16% | 142  (101)  28% | 1161  20% |
| Don’t know Observed  (Expected)  % Within Column | 612  (566)  14% | 54  (58)  12% | 66  (85)  10% | 44  (67)  9% | 776  13% |
| X^2^ = 167, df = 6, P < 0.0001 Total | 4304  100% | 439  100% | 649  100% | 512  100% | 5904  100% |
| Missing values | 657 | 94 | 194 | 95 | 1040 |
| …conditions that are life-threatening and can be prevented |  |  |  |  |  |
| Yes Observed  (Expected)  % Within Column | 4203  (4198)  98% | 425  (428)  97% | 636  (633)  98% | 495  (499)  97% | 5759  98% |
| No Observed  (Expected)  % Within Column | 44  (48)  1% | 7  (5)  2% | 7  (7)  1% | 8  (6)  2% | 66  1% |
| Don’t know Observed  (Expected)  % Within Column | 57  (58)  1% | 7  (6)  2% | 6  (9)  1% | 9  (7)  2% | 79  1% |
| X^2^ = 4, df = 6, P = 0.7 Total | 4304  100% | 439  100% | 694  100% | 512  100% | 5904  100% |
| Missing values | 657 | 94 | 194 | 95 | 1040 |
| …conditions that are serious (but not life-threatening) and cannot be prevented |  |  |  |  |  |
| Yes Observed  (Expected)  % Within Column | 3342  (3210)  78% | 213  (327)  49% | 497  (484)  77% | 351  (382)  69% | 4403  75% |
| No Observed  (Expected)  % Within Column | 540  (677)  13% | 169  (69)  39% | 102  (102)  16% | 118  (81)  23% | 929  16% |
| Don’t know Observed  (Expected)  % Within Column | 422  (417)  10% | 57  (43)  13% | 50  (63)  8% | 43  (50)  8% | 572  10% |
| X^2^ = 247, df = 6, P < 0.0001 Total | 4304  100% | 439  100% | 649  100% | 512  100% | 5904  100% |
| Missing values | 657 | 94 | 194 | 95 | 1040 |
| …conditions that are serious (but not life-threatening) and can be prevented |  |  |  |  |  |
| Yes Observed  (Expected)  % Within Column | 4195  (4187)  98% | 424  (427)  97% | 630  (631)  97% | 495  (498)  97% | 5744  97% |
| No Observed  (Expected)  % Within Column | 51  (61)  1% | 11  (6)  2% | 12  (9)  2% | 10  (7)  2% | 84  1% |
| Don’t know Observed  (Expected)  % Within Column | 58  (55)  1% | 4  (6)  1% | 7  (8)  1% | 7  (7)  1% | 76  1% |
| X^2^ = 8, df = 6, P = 0.2 Total | 4304  100% | 439  100% | 649  100% | 512  100% | 5904  100% |
| Missing values | 657 | 94 | 194 | 95 | 1040 |
| "If I was a research participant, I'd like to receive information that..." |  |  |  |  |  |
| …demonstrates how I might respond to different medications or drugs (e.g. statins, anti-depressants etc) |  |  |  |  |  |
| Yes Observed  (Expected)  % Within Column | 3960  (3956)  94% | 397  (414)  90% | 614  (609)  95% | 485  (476)  96% | 5456  94% |
| No Observed  (Expected)  % Within Column | 123  (133)  3% | 25  (14)  6% | 24  (21)  4% | 12  (16)  2% | 184  3% |
| Don’t know Observed  (Expected)  % Within Column | 126  (120)  3% | 18  (13)  4% | 10  (18)  1% | 11  (14)  2% | 165  3% |
| X^2^ = 19, df = 6, P = 0.003 Total | 4209  100% | 440  100% | 648  100% | 508  100% | 5805  100% |
| Missing values | 752 | 93 | 195 | 99 | 1139 |
| .. tells me if I'm a carrier of a condition that could be relevant to my children |  |  |  |  |  |
| Yes Observed  (Expected)  % Within Column | 3982  (3963)  95% | 388  (414)  88% | 621  (616)  96% | 474  (478)  93% | 5465  94% |
| No Observed  (Expected)  % Within Column | 124  (138)  3% | 33  (14)  8% | 12  (21)  2% | 21  (17)  4% | 190  3% |
| Don’t know Observed  (Expected)  % Within Column | 103  (109)  2% | 19  (11)  4% | 15  (17)  2% | 13  (13)  3% | 150  3% |
| X^2^ = 38, df = 6, P < 0.0001 Total | 4209  100% | 440  100% | 648  100% | 508  100% | 5805  100% |
| Missing values | 752 | 93 | 195 | 99 | 1139 |
| .. is not immediately relevant but could be useful later in life (e.g. relating to a very late onset cancer or predisposition to strokes) |  |  |  |  |  |
| Yes Observed  (Expected)  % Within Column | 3665  (3604)  87% | 322  (377)  73% | 567  (555)  87% | 416  (435)  82% | 4970  86% |
| No Observed  (Expected)  % Within Column | 285  (347)  7% | 83  (36)  19% | 51  (53)  8% | 59  (42)  12% | 478  8% |
| Don’t know Observed  (Expected)  % Within Column | 259  (259)  6% | 35  (27)  8% | 30  (40)  5% | 33  (31)  6% | 357  6% |
| X^2^ = 93, df = 6, P < 0.0001 Total | 4209  100% | 440  100% | 648  100% | 508  100% | 5805  100% |
| Missing values | 752 | 93 | 195 | 99 | 1139 |
| .. is uncertain and cannot be interpreted at the moment |  |  |  |  |  |
| Yes Observed  (Expected)  % Within Column | 1829  (1811)  43% | 139  (189)  32% | 294  (279)  45% | 235  (219)  46% | 2497  43% |
| No Observed  (Expected)  % Within Column | 1677  (1778)  40% | 256  (186)  58% | 278  (274)  43% | 241  (215)  48% | 2452  42% |
| Don’t know Observed  (Expected)  % Within Column | 703  (621)  17% | 45  (65)  10% | 76  (96)  12% | 32  (75)  6% | 856  15% |
| X^2^ = 97, df = 6, P < 0.0001 Total | 4209  100% | 440  100% | 648  100% | 508  100% | 5805  100% |
| Missing values | 752 | 93 | 195 | 99 | 1139 |
| ... is not likely to be of serious health importance (e.g. mild eyesight problems) |  |  |  |  |  |
| Yes Observed  (Expected)  % Within Column | 2911  (2802)  69% | 175  (293)  40% | 430  (431)  66% | 348  (338)  68% | 3864  67% |
| No Observed  (Expected)  % Within Column | 1082  (1195)  26% | 238  (125)  54% | 188  (184)  29% | 140  (144)  28% | 1648  28% |
| Don’t know Observed  (Expected)  % Within Column | 216  (212)  5% | 27  (22)  6% | 30  (33)  5% | 20  (26)  4% | 293  5% |
| X^2^ = 168, df = 6, P < 0.0001 Total | 4209  100% | 440  100% | 648  100% | 508  100% | 5805  100% |
| Missing values | 752 | 93 | 195 | 99 | 1139 |
| .. tells me about my ancestry |  |  |  |  |  |
| Yes Observed  (Expected)  % Within Column | 3599  (3487)  86% | 270  (365)  62% | 526  (537)  81% | 414  (420)  81% | 4809  83% |
| No Observed  (Expected)  % Within Column | 427  (540)  10% | 147  (56)  33% | 97  (83)  15% | 74  (65)  15% | 745  13% |
| Don’t know Observed  (Expected)  % Within Column | 183  (182)  4% | 23  (19)  5% | 25  (28)  4% | 20  (22)  4% | 251  4% |
| X^2^ = 202, df = 6, P < 0.0001 Total | 4209  100% | 440  100% | 648  100% | 508  100% | 5805  100% |
| Missing values | 752 | 93 | 195 | 99 | 1139 |
| Let's assume it is possible to return Incidental Findings relating a condition that is serious and preventable. Does the level of risk of actually getting the condition affect whether you think the result should be returned? "If I was a research participant, I'd like to receive information that predicts..."  ... there is a 1 in 100 risk (i.e. 1% chance) that this condition will occur |  |  |  |  |  |
| Yes Observed  (Expected)  % Within Column | 2465  (2507)  61% | 243  (268)  56% | 445  (379)  69% | 334  (315)  65% | 3487  62% |
| No Observed  (Expected)  % Within Column | 1152  (1148)  28% | 156  (123)  36% | 152  (182)  24% | 137  (144)  27% | 1597  28% |
| Don’t know Observed  (Expected)  % Within Column | 446  (408)  11% | 36  (44)  8% | 47  (65)  7% | 39  (51)  8% | 568  10% |
| X^2^ = 37, df = 6, P < 0.0001 Total | 4063  100% | 435  100% | 644  100% | 510  100% | 5652  100% |
| Missing values | 898 | 98 | 199 | 97 | 1292 |
| ... there is a 10 in 100 risk (i.e. 10% chance) that this condition will occur |  |  |  |  |  |
| Yes Observed  (Expected)  % Within Column | 3150  (3158)  76% | 318  (338)  73% | 533  (501)  83% | 392  (396)  77% | 4393  78% |
| No Observed  (Expected)  % Within Column | 570  (584)  14% | 78  (63)  18% | 80  (93)  12% | 84  (73)  16% | 812  14% |
| Don’t know Observed  (Expected)  % Within Column | 343  (321)  8% | 39  (34)  9% | 31  (51)  5% | 34  (40)  7% | 447  8% |
| X^2^ = 22, df = 6, P = 0.001 Total | 4063  100% | 435  100% | 644  100% | 510  100% | 5652  100% |
| Missing values | 898 | 98 | 199 | 97 | 1292 |
| ... there is a 50 in 100 risk (i.e. 50% chance) that this condition will occur |  |  |  |  |  |
| Yes Observed  (Expected)  % Within Column | 3818  (3819)  94% | 405  (409)  93% | 612  (605)  95% | 478  (479)  94% | 5313  94% |
| No Observed  (Expected)  % Within Column | 129  (132)  3% | 14  (14)  3% | 17  (21)  3% | 23  (17)  4% | 183  3% |
| Don’t know Observed  (Expected)  % Within Column | 116  (112)  3% | 16  (12)  4% | 15  (18)  2% | 9  (14)  2% | 156  3% |
| X^2^ = 7, df = 6, P = 0.3 Total | 4063  100% | 435  100% | 644  100% | 510  100% | 5652  100% |
| Missing values | 898 | 98 | 199 | 97 | 1292 |
| ... there is a 90 in 100 risk (i.e. 90% chance) that this condition will occur |  |  |  |  |  |
| Yes Observed  (Expected)  % Within Column | 3908  (3901)  96% | 418  (419)  96% | 625  (620)  97% | 488  (491)  96% | 5439  96% |
| No Observed  (Expected)  % Within Column | 78  (78)  2% | 7  (8)  2% | 10  (12)  2% | 14  (10)  3% | 109  2% |
| Don’t know Observed  (Expected)  % Within Column | 77  (75)  2% | 10  (8)  2% | 9  (12)  1% | 8  (9)  1% | 104  2% |
| X^2^ = 4, df = 6, P = 0.7 Total | 4063  100% | 435  100% | 644  100% | 510  100% | 5652  100% |
| Missing values | 898 | 98 | 199 | 97 | 1292 |
| Assuming research participants consent, do you think genomic researchers should actively search for Incidental Findings that are not relevant to the research study? |  |  |  |  |  |
| Yes Observed  (Expected)  % Within Column | 1395  (1254)  34% | 62  (134)  14% | 163  (195)  26% | 121  (158)  24% | 1741  31% |
| No Observed  (Expected)  % Within Column | 1827  (2064)  45% | 330  (220)  76% | 374  (321)  59% | 334  (260)  65% | 2865  51% |
| Don’t know Observed  (Expected)  % Within Column | 851  (754)  21% | 42  (80)  10% | 97  (117)  15% | 57  (95)  11% | 1047  18% |
| X^2^ = 229, df = 6, P < 0.0001 Total | 4073  100% | 434  100% | 634  100% | 512  100% | 5653  100% |
| Missing values | 888 | 99 | 209 | 95 | 1291 |

| Table S3: Model fit statistics for latent class models | | | | | |
| --- | --- | --- | --- | --- | --- |
| Latent classes | Log Likelihood | Degrees of freedom | G2 | AIC | BIC |
| 2 | -11081.9 | 20 | 159.1 | 181.1 | 253.5 |
| **3** | **-11007.3** | **14** | **9.9** | **43.9** | **155.8** |
| 4 | -11004.8 | 8 | 5.0 | 51.0 | 202.4 |
| 5 | -11003.1 | 2 | 1.6 | 59.6 | 250.5 |

| Table S4: Item-response probabilities for each latent class (probability of negative response) | | | | | | |
| --- | --- | --- | --- | --- | --- | --- |
| Question | Latent Class 1 | | Latent Class 2 | | Latent Class 3 | |
|  | *Estimate* | *SE* | *Estimate* | *SE* | *Estimate* | *SE* |
| Should incidental findings from genome studies be made available to research participants? | 0.01 | 0.00 | 0.59 | 0.10 | 0.06 | 0.01 |
| If you had the choice to receive information about conditions that are life-threatening and cannot be prevented, would you want to know? | 0.04 | 0.01 | 0.88 | 0.05 | 0.34 | 0.03 |
| If you were a research participant in a whole genome study, would you want to be able to receive all of your raw genomic data? | 0.09 | 0.01 | 0.84 | 0.04 | 0.49 | 0.03 |
| “If I was a research participant, I’d like to receive information that predicts a 1 in 100 risk (i.e. 1% chance) that a serious preventable condition will occur” | 0.10 | 0.01 | 0.86 | 0.05 | 0.49 | 0.03 |
| “If I was a research participant, I’d like to receive information that is uncertain and cannot be interpreted at the moment” | 0.10 | 0.02 | 1.00 | 0.01 | 0.86 | 0.02 |
